# Health care professionals from developing countries report educational benefits after an online diabetes course

**DOI:** 10.1186/s12909-017-0935-y

**Published:** 2017-05-31

**Authors:** Nicolai J. Wewer Albrechtsen, Kristina W. Poulsen, Lærke Ø. Svensson, Lasse Jensen, Jens J. Holst, Signe S. Torekov

**Affiliations:** 1 Department of Biomedical Sciences, Faculty of Health and Medical Sciences, University of Copenhagen, Signe Sørensen Torekov, Blegdamsvej 3B, 2200 Copenhagen, Denmark Denmark; 2 NNF Center for Basic Metabolic Research, Faculty of Health and Medical Sciences, University of Copenhagen, Copenhagen, Denmark; 3 Centre for Online and Blended Learning, Faculty of Health and Medical Sciences, University of Copenhagen, Copenhagen, Denmark

**Keywords:** Massive Open Online Course, MOOC, Medical Education, Continuing education, Health care professionals, Diabetes, Obesity

## Abstract

**Background:**

Medical education is a cornerstone in the global combat against diseases such as diabetes and obesity which together affect more than 500 million humans. Massive Open Online Courses (MOOCs) are educational tools for institutions to teach and share their research worldwide. Currently, millions of people have participated in evidence-based MOOCs, however educational and professional benefit(s) for course participants of such initiatives have not been addressed sufficiently. We therefore investigated if participation in a 6 week open online course in the prevention and treatment of diabetes and obesity had any impact on the knowledge, skills, and career of health care professionals contrasting participants from developing countries versus developed countries.

**Methods:**

52.006 participants signed up and 29.469 participants were active in one of the three sessions (2014–2015) of Diabetes - a Global Challenge. Using an online based questionnaire (nine sections) software (Survey Monkey), email invitations were send out using a Coursera based database to the 29.469 course participants. Responses were analyzed and stratified, according to the United Nations stratification method, by developing and developed countries.

**Results:**

1.303 (4.4%) of the 29.469 completed the questionnaire. 845 of the 1303 were defined as health care professionals, including medical doctors (34%), researchers (15%), nurses (11%) and medical students (8%). Over 80% of the health care participants report educational benefits, improved knowledge about the prevention and treatment therapies of diabetes and furthermore improved professional life and practice. Over 40% reported that their professional network expanded after course participation. Study participants who did not complete all modules of the course reported similar impact as the ones that completed the entire course(*P* = 0.9).

Participants from developing countries gained more impact on their clinical practice (94%) compared to health care professionals from developed regions (88%) (Mean of differences = 6%, *P* = 0.03.

**Conclusions:**

Based on self-reports from course participants, MOOC based medical education seems promising with respect to providing accessible and free research-based education to health professionals in both developing and developed countries. Course participants from developing countries report more benefits from course participation than their counterparts in the developed world.

**Electronic supplementary material:**

The online version of this article (doi:10.1186/s12909-017-0935-y) contains supplementary material, which is available to authorized users.

## Background

Massive Open Online Courses (MOOC’s) are publicly accessible educational resources allowing institutions to provide global education and improve the awareness of their existence and the courses may also help to recruit students. As a consequence MOOC’s have become attractive for the leading universities [1] and may furthermore, as stated by Hew and Cheung, represent a significant step towards democratization of knowledge through online education [2]. Although hundreds of MOOCs exist and millions have participated in these, only a few studies [3–6] exist, which have, in addition to the standardized rating(s) provided by companies hosting the MOOC’s, assessed the (educational) impact of the MOOCs on the course participants’ professional life.

Diabetes and obesity are global health-and socioeconomic burdens affecting more than half a billion humans according to the World Health Organization [7, 8] and there is a growing need for new and better therapies. Education of health care professionals by MOOCs is suggested to promote improved care and treatment of obese patients with diabetes, but it is unknown to what extent open online education actually impacts the skills and clinical practice of health care professionals. We hypothesized that health care professional’s had educational benefits with impact on their clinical practice from a 6 week online course in diabetes and obesity treatment therapies and more pronounced in developing countries. To address this, we investigated the impact of a MOOC-based course, termed “Diabetes – a global challenge” which provides an update of basic and clinical sciences in the field of diabetes and obesity, on the clinical practice of health care professionals by a quantitative approach (self-reporting): a web-based questionnaire.

## Methods

### Structure of the online course “Diabetes - a Global Challenge”

The MOOC Diabetes - a Global Challenge (https://www.coursera.org/learn/diabetes) was offered three times during spring 2014 - fall 2015. The course spans six weeks and consists of reading materials, video lectures and multiple choice questions. “Diabetes - a Global Challenge” is a typical MOOC, characterized by instructor-guided lessons combined with tests and assignments, with optional certification, if the learner achieves a certain level, ascertained by on-line tests. The participants are encouraged to join the online discussions forum together with peers and the instructor in order to reflect and share what they have learned.

### Content of the online course “Diabetes - a Global Challenge”

The MOOC Diabetes – a Global Challenges includes 12 weeks of syllabus (https://www.coursera.org/learn/diabetes#syllabus) including areas like epidemiology, prevention of diabetes, obesity, pharmacological treatment of diabetes, genetic forms of diabetes etc.

### Course participants

52.006 participants signed up and 29.469 participants were active at one of the three courses (during 2014–2015). We defined participants as ‘active’ when they had made a login to the “online course room” and had accessed at least one learning module (lecture videos or assignments). The 29.469 participants were included in our online post-course survey. Among the 29.469 participants 1.303 responded to our survey (4.4%). Health care professionals represented ~64% (*n* = 845) of the total responders and were defined as being either: medical doctor, nurse, midwife, nutritionist, technician, working in the health care industry, health care researcher/academic, medical student or health care student. On average, 77% of the participants from the Cousera based rating(s) (part of the actual course) responded that: ‘*I have learned something I can use in my professional life*’. However, we wanted to explore the demographic and educational background of the health care professionals, and to evaluate the impact of the course on their professional education and practice, and chose to use a web-based questionnaire for this.

### The web-based questionnaire

The post-course questionnaire was prepared using the software package Survey Monkey©. Emails, including a link to the web-based survey, were prepared and sent out (during October 2015) to the participants using the Coursera online software. The primary purpose of our survey was to collect data concerning the participant’s professional benefits from the course contrasting developed vs developing countries. The questionnaire was structured by nine closed-ended questions. The first part (questions 1–8) assessed demographics, level of education and similar. The second part (question 9) consisted of 15 “statements”.

Q1-Q7 and were subdivided into different subtitles based on career or educational benefits.

Q 1: The course has been a useful learning experience.

Q 2: I have gained knowledge for my field of study or work.

Q 3 I have had educational benefit(s).

Q 4: I have learned something I can use in my professional life.

Q 5: I have enhanced my skills for current job.

Q 6: I have increased my professional network.

Q 7: I have collaborated with other students / used discussion forums and Facebook. Additional file 1: Figure S1 shows an example of a statement using rating scales and the “response” options. For the entire questionnaire please see Additional file 2: Data I.

### Data management and statistics

We applied the United Nations Statistics Division system (http://unstats.un.org) to structure and organize demographic data. United Nations Statistics Division group countries into two regions, developed regions and developing regions, respectively. The developing regions are: Africa, the Caribbean, Central America, South America, Asia (excluding Japan) and Oceania (excluding Australia and New Zealand). The developed regions are: Northern America, Europe, Japan, Australia and New Zealand. To compare differences between total population and target population we used unpaired t-test adjusted for multiple testing based on mean of differences (95% confidence interval). *P* < 0.05 was considered significant. Calculations were made using GraphPad Prism version 6.04 for Windows, GraphPad Software, La Jolla California USA, www.graphpad.com and STAT14, Boston, MA, USA. For illustrations we used the Adobe CS6 software suite (California, USA).

## Results

### Demographic data of the participants

There were no significant differences with respect to demographic data (gender, *P* = .45; age, *P* = .76; completion percentages of the course, *P* = 0.81) between health care professionals (*n* = 845) and other respondents (*n* = 1.303). See Additional file 1: Table S1 and S2 for further information. The average age of the target participants (e.g. the health care professionals) was 40 ± 0.4 years with a gender distribution of 45% females. Furthermore, there were no significant differences (*P* > 0.05) in gender and age composition between the developing and the developed group (data not shown).

### Occupation of the health care professionals

**Fig. 1:**
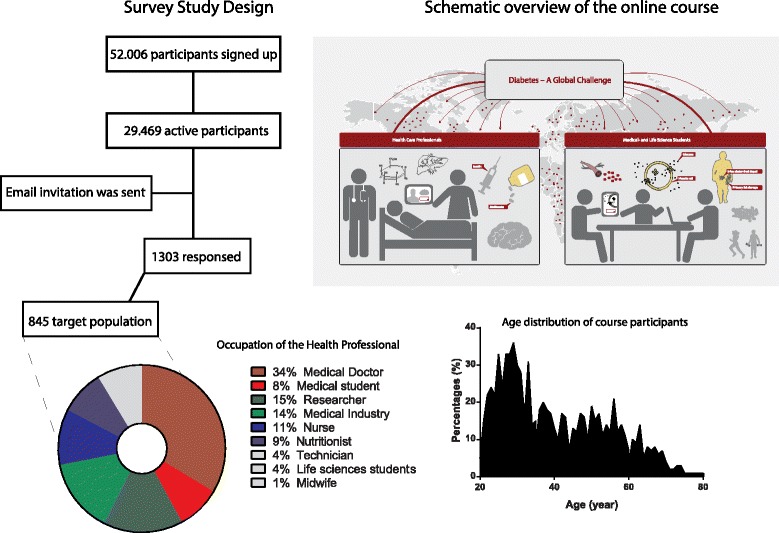
An overview of study design, the participants and the online course

The most frequent occupation in the target population was medical doctors (34%) and the least frequent midwives (0.5%). Most of the students (12% in total) attending the course were medical students (75%). See Fig. 1.

### Health care professionals from developing countries are more likely to report benefits from course participation

In Table 1 responses (given as percentages) from the health care professionals stratified by occupation are listed for the “first part” of the survey which focusses on the participants’ general impression of the course. To the question ‘the course has been an useful learning experience’ (Q1) 94% of participants responded that they “agreed” or “strongly agreed”; 79% “agreed” or “strongly agreed” that ‘I have gained knowledge for their field of study or work’ (Q2); and 81% “agreed” or “strongly” agreed that ‘I have had educational benefits from the course’ (Q3). Interestingly, the responses from the participants depended on the developmental state of their country. Thus, the health care professionals from developing regions were significantly more likely to report that the course was a useful learning experience compared to health care professionals from developed regions(Mean of differences = 8%, *P* = 0.03).

**Table 1:** Health care professionals from developing countries are more likely to report benefits from course participants

	The course has been a useful learning experience			I have gained knowledge for their field of study or work			I have had educational benefits from the course		
Occupation / response	Strongly agree	Agree	Total	Strongly agree	Agree	Total	Strongly agree	Agree	Total
Doctor	49%^a^	48%	98%	32%	59%	91%	30%	54%	84%
Nurse	62%	31%	92%	43%	47%	90%	37%	46%	83%
Midwife	75%	0%	75%	50%	50%	100%	25%	25%	50%
Nutritionist	51%	48%	99%	44%	53%	97%	37%	49%	86%
Technician	46%	51%	97%	19%	49%	68%	16%	70%	87%
Industry	52%	43%	95%	37%	53%	90%	33%	51%	85%
Research/ academic	52%	47%	98%	33%	55%	89%	32%	51%	83%
Med. stud.	54%	41%	94%	39%	45%	85%	35%	51%	86%
Life.Sci. student	47%	50%	97%	34%	56%	91%	35%	51%	86%
Average	54%	40%	94%	37%	59%	79%	31%	50%	81%
Developing *n* = 453	53%	45%	98%	37%	53%	91%	33%	53%	87%
Developed *n* = 392	50%	41%	91%	33%	55%	88%	30%	51%	81%

^a^Numbers are reduced to two significant figures

### Six weeks of online education improve the professional practice in a cohort of 842 health care professional, but the response depends on WHO-based classification of their nationality

The focus of the survey was to assess the impact of the online course Diabetes - a Global Challenge on the participant’s professional career. In Table 2, data are presented from the survey’s main outcomes: 1) whether the participation in the 6 week online course improved their knowledge about diabetes and obesity to be used in their professional life and 2) whether participation provided knowledge that positively affected their current job. On average 89% of the health care professionals reported that they have learned something they can use in their professional lives. Medical doctors, nurses and medical students reported that they, by following the course, had gained new knowledge of diabetes and obesity to be implemented in their professional lives. 96% of these agreed or strongly agreed to the “I have learned something I can use in my professional life” statement. 88% of the nutritionists had enhanced their skills for current job in contrast to 46% of the technicians. Interestingly, more health care professionals from developing regions reported that the course had impact on their clinical practice (94%) compared to health care professionals from developed regions (88%) (Mean of differences = 6%, *P* = 0.03).

**Table 2:** Health care professionals improve their professional practice after a 6 week online course

	I have learned something I can use in my professional life			I have enhanced my skills for current job		
Occupation / response	Strongly agree	Agree	Total	Strongly agree	Agree	Total
Doctor	42%^a^	57%	95%	33%	54%	87%
Nurse	54%	42%	96%	40%	43%	83%
Midwife	75%	0%	75%	75%	0%	75%
Nutritionist	53%	43%	96%	32%	56%	88%
Technician	27%	49%	76%	16%	30%	46%
Industry	41%	50%	90%	32%	42%	74%
Research/ academic	46%	46%	92%	23%	51%	74%
Med. stud.	48%	47%	94%	31%	54%	85%
Life.Sci. student	31%	56%	88%	19%	63%	81%
Average	46%	43%	89%	33%		7%
Developing *n* = 453	45%	49%	94%	30%	53.6%	84%
Developed *n* = 392	40%	48%	88%	31%	45.2%	76%

*Numbers are reduced to two significant figures

### Health care professionals increase their professional network during a 6 week online course

The final part of the survey investigated whether and how participants increased their professional network (based on self-reporting) by interaction with other participants and non-participating colleagues (Table 3). 48% of the health care professionals reported that they increased their professional network during the 6 week course. On average 36% of course participants “strongly agreed” or “agreed” to “I have collaborated with other students / used discussion forums and Facebook“. Only 1/3 of the participants had interaction with other course participants thus this may be influenced by a Facebook group developed during “session two” as there were no significant differences between course participants from the session one, session two or session three (*P* = 0.57). Interestingly, the course result with respect to an expanded professional network and interaction with fellow participants depended on the nationality of the participant. Health care professionals from developing regions were more likely to report that they experienced an expanded professional network and collaboration with fellow students compared to health care professionals from developed regions(Mean of differences = 22%, *P* = 0.01).

**Table 3:** Health care professionals increase their professional network after a 6 week online course

	I have increased my professional network			I have collaborated with other students / used discussion forums and Facebook		
Occupation / response	Strongly agree	Agree	Total	Strongly agree	Agree	Total
Doctor	17%^a^	40%	56%	13%	29%	42%
Nurse	17%	29%	45%	12%	30%	42%
Midwife	25%	25%	50%	25%	0%s	25%
Nutritionist	19%	41%	60%	6%	41%	47%
Technician	5%	32%	38%	5%	38%	43%
Industry	14%	33%	47%	10%	16%	25%
Research/ academic	15%	29%	43%	9%	28%	36%
Med. stud.	16%	30%	45%	13%	24%	37%
Life.Sci. student	16%	31%	47%	13%	19%	31%
Average	16%	32%	48%	12%	25%	36%
Developing *n* = 453	19%	41%	60%	13%	34%	43%
Developed *n* = 392	12%	27%	38%	8%	24%	32%

^a^Numbers are reduced to two significant figures

### Subgroup differences between course “completers” and “non-completers”

Table 4 illustrates differences in response to the three survey question 1) “the course has been a useful learning experience”, 2) “I have learned something I can use in my professional life” and 3)“I have collaborated with other students / used discussion forums and Facebook” between health care professionals who completed one of the three session of the course (termed “completers”) and health care professionals who did not complete one of the three session of the course (termed “non-completers”). The majority (89%) of the completers reported both career and professional related impact outcomes of the course. Importantly, and unexpected, similar overall trends (educational benefits, professional network) were observed in group of non-completers. However, the non-completer subgroup did not respond as positively as the completer subgroup (78% versus 92%) with regards to the course impact on the participant’s professional life.

**Table 4:** Subgroup differences between course “completers” and “non-completers”

	The course has been a useful learning experience		I have learned something I can use in my professional life		I have collaborated with other students / used discussion forums and Facebook	
Occupation / response	Completers	Non-completers	Completers	Non-completers	Completers	Non-completers
Doctor	99%^a^	96%	95%	95%	94%	86%
Nurse	96%	87%	98%	92%	90%	97%
Midwife	75%	0%	75%	0%	75%	0%
Nutritionist	100%	97%	97%	94%	95%	80%
Technician	100%	95%	82%	70%	53%	40%
Industry	96%	94%	93%	87%	73%	75%
Research/academic	100%	97%	94%	91%	70%	78%
Med. stud.	100%	90%	100%	90%	84%	85%
Life.Sci. student	100%	95%	92%	84%	85%	79%
Average	96%	84%	92%	78%	80%	69%

^a^Numbers are reduced to two significant figures

## Discussion

Health care professionals may improve their knowledge in diabetes after a 6 week online course. A major limitation of our study is a low response rate of ~5% since we only report response from course participants and not actually learning we cannot conclude if the participating health care professionals had increased their overall learning [9]. Over 90% of the 287 medical doctors responded that they had improved their knowledge of diabetes to be used in their professional life and importantly felt that the course had enhanced their professional skills related to work. The majority (89%) of the health care professionals who completed the course reported that they gained knowledge both related to future career and educational benefits. This suggest that MOOCs, designed with an appropriate pedagogical foundation [10], may improve learning outcomes as also reported by Glance et al. [11]. Surprisingly, the health care professionals who did not complete the course reported similar trends. Even though the percentages among completers were high in general, 77% of the non-completers still reported that they had gained career and educational benefits. Thus we conclude that non-completers also benefit from the course despite not completing it. These tendencies reported here align to what was reported by other MOOC in mathematics [12]. It has been suggested that MOOCs may expand educational outcomes between course participant from different socioeconomic groups. In the current study we have no socioeconomically data but we do have country of origin and find that participants from developing countries benefit more in clinical practices compared to developed countries. And health care professionals in developing regions reported higher impact measures with regards to educational and career benefits, which may be related to the statements from the participants from developing countries on having less access to state-of the art research in diabetes and obesity and as well new treatment therapies [13]. In addition, the positive aspects of participating in the course highlighted by an increased professional network and interaction with fellow participants is similar to what was reported in a small randomized controlled trial [6]. That said, it is important to acknowledge that changing a person’s knowledge does not necessarily translate into their practice or behavior [14]. As our study do not provide data on the actually measured impact on the course participants we refrain from concluding the extent of the impact on their professional life other than the self-reported. Another limitation of the current study is the inability to accurately investigate the real extent on the course participants’ competences as our data are based on self-assessments and not measured objectively [15]. Therefore, future studies may include objective assessment of outcome(s) such as the impact of the MOOC on the physician’s professional life. Furthermore, a relatively low response rate and the sole reliance on self-reports may influence the data reported here.

Medical education, including basic and clinical sciences, remains a ground pillar in clinical medicine. Diabetes and obesity are interconnected diseases affecting more than 500 million people globally and increase overall mortality rates, with cardiovascular mortality increasing by more than 200%. A conflict exists for clinicians and other health care professionals, who wish (or are obliged) to continue and maintain their medical expertise through continuing education [16] but cannot find time to locate and evaluate the thousands of terabytes of digital, online-available resources including MOOCs [17], and therefore refrain from continuation of postgraduate education. Technological advances during the last decade have provided several novel opportunities for continuation of postgraduate medical education. Online resources, including but not limited to MOOCs, may serve as substantial supplements to current post-graduate education [18] and to keep the health care professionals updated on new treatment therapies and guidelines. MOOC is an online teaching platform, where everyone with an internet connection, regardless of location, can enroll in a course in their field of interest. However, the impact of such online resources and courses has not been investigated thoroughly [19].

To improve the value of our survey we included all the participants in the questionnaire request send out by email, regardless of whether they had or had not completed the course, from a hypothesis that the benefit would not necessarily depend on completion of the entire course. The health care professionals (the target population) were considered to be representative compared to the general population, as they did not differ significantly (*P* = 0.76) from the entire population of 29.469 participants with regards to the demographics data or to their overall course rating sampled randomly during the three sessions of the course. In addition, the study applied the statistic randomization method termed simple random sampling (each participant of the total population has an equal probability of being chosen), however as the survey data are self-reported the external validity of this survey, and hence the generalizability to the entire population of health care professionals, may suffer. To avoid missing data we designed the survey so it was mandatory to complete the entire questionnaire before uploading the data to the database (survey monkey), and as a consequence responders that did not do that were not included in the final analysis. In addition, participants enrolled in the 3 different sessions may have had varying experiences as the course developed which may have affected the result - however, as there were no significant differences between responses from participants in session one, two and three we do not find this as a major limitation of the current study. Based on the current study design, sampling bias may not necessarily be a major concern, as explained previously, but recall bias and especially response bias may in theory have effects on the internal validity of our survey. Undoubtedly, the data from the survey are influenced by selection bias which we cannot control for in the current setup; however, given that survey participants and course participants did not differ significantly with regards to nationality, age, gender and completion rates we believe that the external validity of the current study is adequate for the conclusions made.

## Conclusion

In summary, our study indicates that a 6 week internet-based course in diabetes and obesity treatment may serve as an important resource in postgraduate education for medical doctors as well other health care professionals. In a wider perspective, MOOC based education may assist the professional community to improve global health and medical education by providing the latest evidence-based guidelines and state of the art research in an easily accessible and globally available way to providers of clinical care of diabetes and obesity.

## Additional files

Additional file 1: Figure S1. and **Table S1.** and **S2.** **Figure S1.** Shows how questions were used in the survey. **Table S1.** Shows the age distribution of the study participants. **Table S2.** Shows the regional distribution of the study participants. (DOCX 31 kb)

Additional file 2: Data 1. The Questionnaire used for the study participants. The questionnaire consist of nine questions and was distributed to learners of the cousera-based course Diabetes a global challenge. (PDF 151 kb)

## Abbreviation

MOOC: Massive open online course
