# Supplementary material for: Health care professionals from developing countries report educational benefits after an online diabetes course
Source: BMC Med Educ. 2017 May 31;17:97. doi: 10.1186/s12909-017-0935-y (PMC5452380; doi:10.1186/s12909-017-0935-y)
Supplement: Supplementary file 1 — and Table S1. and S2. Figure S1. Shows how questions were used in the survey. Table S1. Shows the age distribution of the study participants. Table S2. Shows the regional distribution of the study participants. (DOCX 31 kb) [file 12909_2017_935_MOESM1_ESM.docx]

**Supplementary Figure 1**

Figure 1 shows the question ‘The course has been an useful learning experience’ that was asked to the participants in the survey.


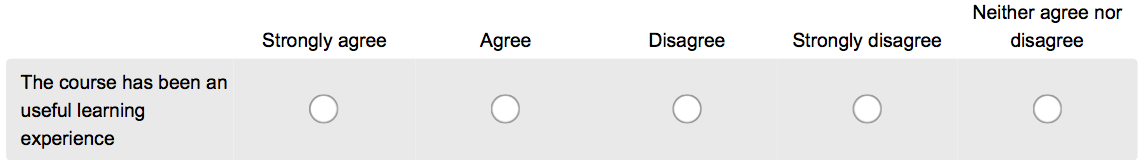


**Supplementary Table 1**

Age:

55% (n=465) of the health care professionals were 40 years old or younger in contrast to all respondents in which 47% (n=397) were 40 years old or younger. This shows a difference of 8% between these two groups (P=0.02). Regarding the respondents who are 60 years or older once a difference emerges between the two groups.  The percentages are 12 % and 18 % respectively.

| All | Health Care professional |
| --- | --- |
| 15-29 year: 24,7 %  30-39 year: 22,3 %  40-49 year: 15,6 %  50-59 year:19,1 %  60->80 year: 18,3 % | 15-29 year: 29.6 %  30-39 year: 25.4 %  40-49 year: 15.5 %  50-59 year: 17.3 %  60->80 year: 12.1 % |

**Supplementary Table 2** Table 2 provides information about from which region the respondents live in. As shown in the table, 53.6% (n=453) of the health care professionals live in a developing region, in contrast to 48.3% of all respondents.

| All (1.303) | Health care professional (845) |
| --- | --- |
| Developing regions: 48.3% (n=629)  Developed regions: 51.7% (n=674) | Developing regions: 53.6% (n=453)  Developed: 46.4% (n=392) |
