# Supplementary material for: Health care professionals from developing countries report educational benefits after an online diabetes course
Source: BMC Med Educ. 2017 May 31;17:97. doi: 10.1186/s12909-017-0935-y (PMC5452380; doi:10.1186/s12909-017-0935-y)
Supplement: Supplementary file 2 — The Questionnaire used for the study participants. The questionnaire consist of nine questions and was distributed to learners of the cousera-based course Diabetes a global challenge. (PDF 151 kb) [file 12909_2017_935_MOESM2_ESM.pdf]

Legends for Supplementary Data 1

The appendix shows the questionnaire used for the survey and consist of 9 question. The survey was distributed to learners of the Cousera based MOOC course: Diabetes a Global Challenge.

UNIVERSITY OF COPENHAGEN
FACULTY OF HEALTH AND MEDICAL SCIENCES

Diabetes - a Global Challenge

Welcome to our survey!

Please complete this survey to provide us with valuable feedback regarding the course Diabetes - a Global Challenge.

Thank you!

UNIVERSITY OF COPENHAGEN
FACULTY OF HEALTH AND MEDICAL SCIENCES

Diabetes - a Global Challenge

* 1. Your gender?

* 2. Your age?

* 3. Which course(s) did you attend?

- [ ] Spring 2014
- [ ] Fall 2014
- [ ] Spring 2015

* 4. Did you complete the course(s)?

- ◯ Yes
- ◯ No
- ◯ Some

* 5. Where do you live?

[ ]

* 6. Primary interest in diabetes?

[ ]

* 7. What best describes your highest level of education?

[ ]

* 8. Primary occupation?

[ ]

* 9. How do you agree in the following statements?

	Strongly agree	Agree	Disagree	Strongly disagree	Neither agree nor disagree
The course has been an useful learning experience	◯	◯	◯	◯	◯
I have learned something I can use in my professional life	◯	◯	◯	◯	◯
I have enhanced my skills for current job	◯	◯	◯	◯	◯
I am now better qualified to apply for new jobs	◯	◯	◯	◯	◯
The course has had influence on finding a new job/starting my own business	◯	◯	◯	◯	◯
The course has had influence on me getting a pay increase or promotion	◯	◯	◯	◯	◯
I have advanced my career through this course	◯	◯	◯	◯	◯

	Strongly agree	Agree	Disagree	Strongly disagree	Neither agree nor disagree
I have increased my professional network	◯	◯	◯	◯	◯
I have gained knowledge for my field of study or work	◯	◯	◯	◯	◯
I have collaborated with other students / used discussion forums and Facebook	◯	◯	◯	◯	◯
I have had educational benefit(s)	◯	◯	◯	◯	◯
The course has contributed to gaining credits or prerequisites for academic degree/program	◯	◯	◯	◯	◯
The course helped me decide what to study	◯	◯	◯	◯	◯
I have considered applying to University of Copenhagen	◯	◯	◯	◯	◯
I would recommend this course to friends and collegues	◯	◯	◯	◯	◯

Thank you for your participation!
